# Trends in COVID-19 Health Disparities in North Carolina: Preparing the Field for Long-Haul Patients

**DOI:** 10.3390/healthcare9121704

**Published:** 2021-12-08

**Authors:** Thais Muratori Holanda, Claudia Alberico, Leslimar Rios-Colon, Elena Arthur, Deepak Kumar

**Affiliations:** Julius L. Chambers Biomedical Biotechnology Research Institute, North Carolina Central University, Durham, NC 27707, USA; tmurator@nccu.edu (T.M.H.); coliveir@nccu.edu (C.A.); lrioscolon@nccu.edu (L.R.-C.); earthur1@nccu.edu (E.A.)

**Keywords:** COVID-19, health disparity, long-COVID, persistent, symptoms

## Abstract

Long-term coronavirus disease 2019 (long-COVID) refers to persistent symptoms of SARS-CoV-2 (COVID-19) lingering beyond four weeks of initial infection. Approximately 30% of COVID-19 survivors develop prolonged symptoms. Communities of color are disproportionately affected by comorbidities, increasing the risk of severe COVID-19 and potentially leading to long-COVID. This study aims to identify trends in health disparities related to COVID-19 cases, which can help unveil potential populations at risk for long-COVID. All North Carolina (NC) counties (*n* = 100) were selected as a case study. Cases and vaccinations per 1000 population were calculated based on the NC Department of Health and Human Services COVID-19 dashboard with reports current as of 8 October 2021, which were stratified by age groups and race/ethnicity. Then, NC COVID-19 cases were correlated to median household income, poverty, population density, and social vulnerability index themes. We observed a negative correlation between cases (*p* < 0.05) and deaths (*p* < 0.01) with both income and vaccination status. Moreover, there was a significant positive association between vaccination status and median household income (*p* < 0.01). Our results highlight the prevailing trend between exacerbated COVID-19 infection and low-income/under-resourced communities. Consequently, efforts and resources should be channeled to these communities to effectively monitor, diagnose, and treat against COVID-19 and potentially prevent an overwhelming number of long-COVID cases.

## 1. Introduction

As of 11 October 2021, the World Health Organization (WHO) reported 237,196,253 confirmed cases of SARS-CoV-2 (COVID-19) globally, including 4,840,189 deaths directly attributed to this disease. During this same period in the United States, there have been 43,792,254 confirmed cases of COVID-19 with 703,599 deaths [1]. The immediate epidemiological, clinical, and pathophysiological consequences for patients with COVID-19 (acute COVID-19) have been rigorously reported. However, the effects of the long-term sequelae of the disease remain unclear. A study estimated between 10% and 30% of people who have been infected with COVID-19 develop prolonged symptoms [2]. Long-term coronavirus disease 2019 (long-COVID) has been defined as health problems related to COVID-19 that persist after four weeks from the date of infection, deeply impairing the individual’s quality of life [3,4].

Millions of COVID-19 infections worldwide, with numbers yet rising, point to a potentially dramatic increase in the number of patients suffering from lingering symptoms (often referred to as “long-haulers”) [5]. Long-haulers report protracted illness affecting a variety of systems: neurocognitive (e.g., dizziness, confusion, loss of attention), autonomic (e.g., tachycardia, chest pain, palpitations), gastrointestinal (e.g., diarrhea, vomiting, abdominal pain), respiratory (e.g., fatigue, cough, throat pain), musculoskeletal (e.g., pain), psychological (e.g., post-traumatic stress, anxiety, insomnia), and other (e.g., skin rashes, loss of smell and taste) [6]. Recovery from COVID-19 is multifactorial and heterogeneous. Factors such as age and sex, and comorbidities such as obesity, diabetes, chronic obstructive pulmonary disease (COPD), cardiovascular diseases (CVD), hypertension, asthma, etc., modulate recovery, further complicating the pathophysiology of the disease and promoting long-COVID [7].

Social determinants of health such as neighborhood and physical environment, job conditions, healthcare access, education, and income may contribute to health disparities in minority groups. This increases the risk of COVID-19 exposure, infection rate, hospitalization, long-term health, social consequences, and death [8]. For example, findings suggest that racial minorities are disproportionately affected by comorbidities that put them at risk of developing severe COVID-19 symptoms [9].

Thus, this study uses datasets from the North Carolina Department of Health and Human Services (NCDHHS) to determine correlations between social determinants of health and health disparities in COVID-19 cases. This may help recognize potential communities that should better prepare for long-COVID syndrome. Understanding these disparities can guide the allocation of regional, national, and global public health resources to manage COVID-19 long-term symptoms and to protect vulnerable populations.

## 2. Materials and Methods

### 2.1. Setting and Population

North Carolina (NC) was selected as a case study. The first case of COVID-19 infection was reported in NC on 3 March 2020. Reports from 8 October 2021 showed 1,425,062 cases and 17,104 COVID-related deaths in this state [10]. This state readily provides access to available COVID-19 data through the NCDHHS [11]. The NCDHHS provides the number of weekly COVID-19 cases, deaths, and vaccination status that can be associated with demographics via the COVID-19 Dashboard [11]. Data are provided by county and stratified by race/ethnicity and age. Data for any county with a population of 500 or fewer were suppressed for privacy, preventing targeting or exposure of populations. If only one sub-group with a population of 500 or fewer required suppression, the next smallest subgroup was also suppressed for privacy. The subgroups for age and race/ethnicity were managed to fit the same categories as the US Federal Census data. All COVID-19 cases and deaths were calculated per 100,000 inhabitants for each county. For each demographic group (age under 18 years old, age between 18 and 64 years old, race/ethnicity White, race/ethnicity Black, etc.), cases were calculated per 1000 inhabitants. Arithmetic density (population per square mile) was calculated based on population per county and its total area in square miles.

### 2.2. Study Design

The American Community Survey (ACS) 5-year estimates for 2019 [12] were downloaded in August of 2021 with data per NC county. Population demographics of interest were total population, population per age (under 18 years old, between 18 and 64 years old, and 65 years old or more), population per race (Black, Asian, White, and Other), and population per ethnicity (Hispanic and non-Hispanic). County characteristics were determined by median household income, percent population in poverty, population density, and social vulnerability.

The Social Vulnerability Index (SVI) [13] was calculated using the same ACS estimates and determined by themes. Each theme comprises a set of variables from the population and are as follows: socioeconomic status (below poverty, unemployed, income, no high school diploma), household composition and disability (aged 65 or older, aged 17 or younger, civilian with a disability, single-parent households), minority status and language (minority, aged 5 or older who speaks English “less than well”), and housing type and transportation (multi-unit structures, mobile homes, crowding, no vehicle, group quarters). The sum of the four items comprises the overall vulnerability index. The index ranges from zero to one, with “0” being the lowest level of vulnerability and “1” being the highest level of vulnerability. That is, a community with an SVI of 0.3 would have better outcomes from a vulnerable situation (such as a pandemic) than a community with a score of 0.8, for example.

### 2.3. Statistical Analysis

The analytical approach was to seek correlations between the number of cases and vaccinations per 1000 population and the sociodemographic characteristics of the county. Due to lack of normality in data distribution, a non-parametric bivariate correlation test was used. A two-tailed test was performed, and the Spearman correlation coefficient was used to identify significant associations. Statistical analyses were performed using the Statistical Package for the Social Sciences (SPSS) (IBM Corp. Released 2019. IBM SPSS Statistics for Windows, Version 26.0. Armonk, NY, USA: IBM Corp.), and the level of significance was maintained at 5%. Nonetheless, SPSS outputs identified the level of significance at 5% and 1%, which is the reason why both are noted in the tables.

## 3. Results

**Figure 1:**
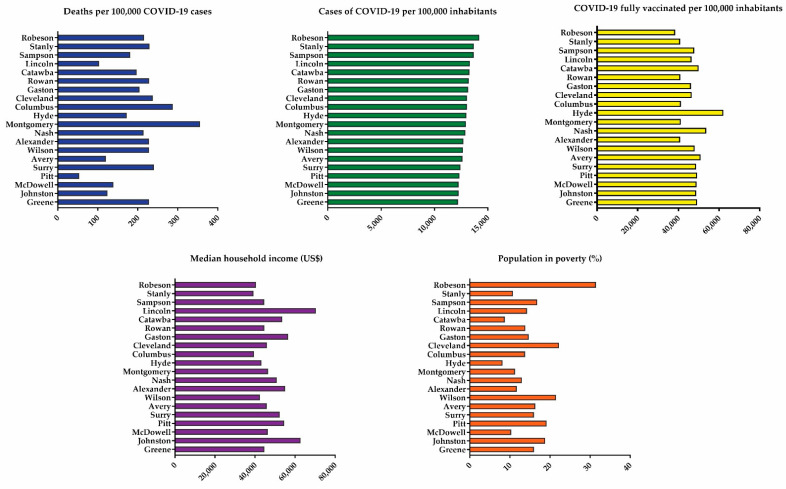
Distribution for the 20 counties of North Carolina with the highest incidence of COVID-19 cases.

NC counties have a widespread population, ranging from 4095 to 1,074,475. Over 75% of the population is between the ages of 18 and 64, with an older population (65+) of 17%. County population, sociodemographic characteristics, and socioeconomic status can be found in Table 1. The distribution summary of COVID-19 cases per county for the top 20 counties in North Carolina can be found in Figure 1 (data for all 100 counties available in the Supplementary Table S1).

Analysis of data from 20 counties in NC (Figure 1) shows no evident trend correlating the number of COVID-19 cases with the number of deaths, median household income, and poverty. Notably, Robeson county had the highest rate of cases (14,209 per 100,000 inhabitants) and the highest percentage of poverty (31.5%). Additionally, there is a negative correlation between number of cases (*p* < 0.05) and number of deaths (*p* < 0.01) with both income and vaccination (Table 2), showing that the lower the median household income and vaccination status, the higher the number of COVID-19 cases and deaths. Moreover, there is a positive association between the vaccinated population and median household income (Table 2).

With data from the ACS 5-year estimates [12], we sought potential trends in COVID-19 cases in light of the disparities highlighted by this pandemic in NC. This is especially important since mechanisms to determine populations susceptible to lingering symptoms of the disease are yet to be identified. Table 3 presents correlations between cases and vaccination rates per demographic and the ACS estimates for the total population by age, race/ethnicity, household income, and poverty level.

We found a positive association with races other than White, Black, or Asian when correlating with poverty (Table 3). Additionally, there was a strong association between lower income and increased case rate in individuals aged 65 or older. As expected, population density was correlated to a higher rate of cases among most ages (18 years old and older), races, and ethnicity (Asian, Black, White, and Hispanic or Latino).

Analysis of overall SVI demonstrated that increased vulnerability is associated with a higher rate of COVID-19 cases among all ages and those self-identified as White. Nevertheless, when stratifying by socioeconomic status, this association is persistent only in people aged 18 and older (same for all vulnerability themes) and in the number of cases among the White population, but a decrease is seen in the Asian population. The SVI theme 3 (Minority status and language) was associated with Asian and Black races. Theme 4 (Housing type and transportation) was associated with increased COVID-19 cases among Black populations.

Median household income was positively correlated with vaccinations in all ages and Asian, Black, and White race/ethnicity groups. Increased overall SVI lowered the incidence of vaccinations for all age groups, White populations, those identified as “Other” race, and Hispanic or Latino ethnicity. A positive association was found between SVI theme 3 (Minority status and language) and vaccinations for the population aged 18–64 years old and the populations self-identified as Asian and Black.

## 4. Discussion

COVID-19 has amplified health inequalities, highlighting the disproportionate burden experienced by historically marginalized populations. This study showed a higher number of COVID-19 cases and lower number of fully vaccinated persons in counties with lower median household income. Other research has shown that those living in low-income households have an increased risk of illness related to COVID-19 when compared to their counterparts living in high-income households [14].

However, social vulnerability is not entirely dependent on economic status [13]. Its index, SVI, was correlated with an increased rate of COVID-19 cases and lower rate of vaccinations, even when stratified by SVI themes. This is an indication that disease transmission can be affected by other aspects of social vulnerability, such as household composition or the types of occupation prevalent in that community. For example, being employed by a business considered essential and working despite stay-at-home orders leaves employees at a higher risk for exposure. Moreover, living in a crowded, multigenerational household may increase the likelihood of being infected with COVID-19 and limit options to quarantine the affected family members [15].

Long-term symptoms are associated with social determinants, including poverty [16]. It is essential to understand that social inequalities, especially those of financial nature, are even more likely to occur as long-COVID becomes more prevalent. This is because the lingering symptoms affecting a wide range of physical and mental activities can limit patients’ ability to work [17], reducing household income. Long-COVID symptoms in marginalized populations may require similar initiatives as those related to COVID-19 testing and vaccinations, where community partners need to be engaged to provide assistance [18].

Much progress has been made in both vaccine and therapeutic approaches to curb the morbidities and mortality associated with COVID-19 [19]. As of August 2021, over 361 million vaccine doses have been administered in the United States according to the WHO [1], with over 10.4 million of those doses administered in NC [20]. Vaccines have been demonstrated to be highly effective in preventing infection even among newer virus variants [21]. Furthermore, a study by Antonelli et al. demonstrated that vaccines were highly effective in preventing disease and significantly reduced the risk of suffering long-COVID even in patients with breakthrough infections [22].

A smaller study reported that among 1497 fully vaccinated healthcare workers, 39 individuals were confirmed to have COVID-19 breakthrough infections. Of those, 19% reported having long-COVID symptoms six weeks after their initial diagnosis. Their symptoms included loss of smell, coughing, fatigue, difficulty breathing, and weakness. Notably, the majority of the breakthrough infections were mild or asymptomatic, and none required hospitalization [23]. These studies indicate that vaccinated individuals are less likely to develop symptomatic COVID-19 or prolonged symptoms.

Even though the safety and efficacy of vaccines to prevent or ameliorate disease has been thoroughly demonstrated, vaccine hesitancy is still a significant hurdle that undermines the efforts to decrease COVID-19 infection rates and mortality [24]. Although the reasons for this hesitancy are multifactorial, studies indicate that the main drivers at a personal level are confidence, complacency, convenience (or constraints), risk calculation, and collective responsibility [25,26]. Other factors including race, age, gender, income, ethnicity, access to healthcare, religious and/or political affiliation, and mistrust in the scientific establishment further complicate this scenario [27].

Hesitancy is greater among racial and ethnic minorities, aggravating the existing disparities in COVID-19 infection rates and deaths within these communities [28,29]. For example, in a study by Nguyen et al., Black participants living in the US were less likely to be vaccinated than White participants [29]. This hesitancy in communities of color could be explained by past and present experiences and distrust of the healthcare and criminal systems, lack of adequate access to regular healthcare, transportation and language barriers, and the disproportionate burden of this pandemic [28,29]. A recent study of attitudes related to vaccination among racial minority and marginalized populations across nine counties in NC [30] found that factors associated with hesitancy in multivariable logistic regression included being female, being Black, calendar month, safety concerns, and government distrust [30]. To address vaccine hesitancy, particularly among minority populations, a multifaceted strategy prioritizing community engagement and considering people’s experiences must be employed [28]

To effectively control an outbreak and stop the spread of the disease, a patient must be identified and isolated promptly. These efforts are hindered by health insurance coverage gaps, which often prevent a patient from seeking timely medical help due to financial concerns incurring delays in diagnosis and adequate treatment [31]. Lack of healthcare coverage endangers the individual and the community, since a disease can undergo undetected and unchecked increasing transmission rates [32]. According to the US Census Bureau, in 2020, 28.0 million individuals did not have access to any health insurance at any point during the year [33].

Moreover, it has been reported that over 78 million individuals do not have adequate healthcare coverage, putting an even more significant percentage of the population at risk [31]. In addition, given that health insurance coverage is often linked to employment status, millions are at risk of losing their coverage due to the increasing rates of unemployment [31]. Disparities in treatment and outcomes for uninsured patients have been documented for multiple diseases and medical conditions, and they can be even more significant than disparities due to race alone. These disparities may stem from multiple factors such as challenges to access appropriate care, decreased health outcomes, and differences in the quality of care [34]. A report by Families USA uncovered that health insurance gaps were linked to more than 40% of infections and one out of three COVID-19 deaths. This report also revealed that between the start of the pandemic and August 31, 2020, a lack of appropriate health insurance was associated with 2.6 million COVID-19 cases (about 44% of all infections) and 58,000 deaths [32].

Currently, there are no specific drugs to treat COVID-19, although there are treatment options available to reduce virus proliferation such as antivirals (remdesivir), antibodies (tocilizumab), and enzyme inhibitors (fostamatinib); or to alleviate the symptoms such as anti-inflammatories (corticosteroids) and anti-coagulants, among others [19]. Specifically regarding long-COVID symptoms, some pre-existing medicines, such as antihistamines, have been repurposed to treat common long-term disorders such as kidney, thyroid, and gut dysfunctions, depression, and inflammation. There are also advanced ongoing clinical trials focused on treating respiratory conditions for both acute and long-haul symptoms [17].

## 5. Conclusions

Since cases of lingering symptoms have not yet been attributed to a particular biological cause, it is expected that the cases of long-haul symptoms for COVID-19 may follow a trend similar to that of the general infection by the virus causing the disease, with some protection offered by vaccination. This study suggests a trend correlating contamination in low-income and under-resourced communities. The communities at risk for a higher number of cases and, potentially, a higher number of long-haulers can use these results to prepare facilities, medications, and adequate management to better serve patients. Efforts and resources should be allocated to treat the lingering symptoms adequately. Finally, primary care policies need to be strengthened, since health providers are in an optimal position to provide and coordinate care for those patients with higher social vulnerability.

## Figures and Tables

**Table 1:** Description of 100 North Carolina counties according to population, sociodemographic characteristics, and socioeconomic status based on the ACS 5-year Estimates for 2019 [12].

North Carolina Counties (*n* = 100)		Mean	SD
Total Population (per county)		102,648.8	166,430.8
Population per square mile (per county)		201.0	287.3
		Median	IQR
Total Population		55,496.5	97,196
Sociodemographic characteristics (%)		Mean	SD
Age	Under 18	20.8	2.8
	18–64	59.7	3.3
	65 and over	19.5	4.7
Race/Ethnicity	Black	20.3	16.3
	Asian	1.2	1.4
	White	72.2	17.7
	Other	2.2	1.6
	Hispanic or Latino	7.3	4.1
Socioeconomic status			
Median household income (US Dollars)		51,167.35	9116.60
	% in poverty	15.9	4.6

Note: SD = standard deviation; IQR = interquartile range.

**Table 2:** Correlation coefficient between COVID-19 distribution for North Carolina counties (*n* = 100).

	Cases per 100,000	Deaths per 100,000	Vaccination per 100,000
Percent of population in poverty	0.005	0.189	−0.027
Median household income	−0.209 *	−0.386 **	0.323 **
Vaccination per 100,000	−0.233 *	−0.386 **	

* Correlation is significant at the 0.05 level (two-tailed); ** Correlation is significant at the 0.01 level (two-tailed).

**Table 3:** Correlation coefficient between demographics of cases and fully vaccinated individuals per 1000 population and county characteristics in North Carolina counties (*n* = 100).

	Cases per 1000 Population								Vaccines per 1000 Population							
	Age			Self-Identified Race				Ethnicity	Age			Self-Identified Race				Ethnicity
County Characteristics	Under 18 yo	18–64 yo	65 yo or More	Asian	Black	White	Other	Hispanic or Latino	Under 18 yo	18–64 yo	65 yo or More	Asian	Black	White	Other	Hispanic or Latino
Population in poverty (%)	0.044	−0.021	0.063	−0.079	0.013	−0.141	0.211 *	0.030	−0.088	−0.072	−0.039	−0.097	0.003	−0.032	0.021	0.006
Median household income	−0.058	−0.167	−0.278 **	0.180	0.073	−0.164	−0.137	−0.012	0.409 **	0.324 **	0.386 **	0.248 *	0.200 *	0.321 **	0.109	0.198 *
Population density (pop/sq mile)	0.162	0.460 **	0.246 *	0.641 **	0.580 **	0.235 *	−0.156	0.432 **	0.685 **	0.412 **	0.473 **	0.697 **	0.449 **	0.249 *	−0.340 **	0.427 **
SVI ^a^	0.198 *	0.422 **	0.505 **	−0.012	0.161	0.270 **	0.073	0.127	−0.435 **	−0.338 **	−0.258 **	−0.135	−0.133	−0.377 **	−0.211 *	−0.280 **
SVI theme 1Socioeconomic status	0.173	0.291 **	0.458 **	−0.245 *	−0.072	0.235 *	0.168	0.006	−0.621 **	−0.496 **	−0.459 **	−0.395 **	−0.301 **	−0.498 **	−0.095	−0.393 **
SVI theme 2Household composition and disability	0.113	0.263 **	0.473 **	−0.152	0.018	0.190	0.155	0.038	−0.538 **	−0.473 **	−0.358 **	−0.302 **	−0.143	−0.508 **	−0.127	−0.331 **
SVI theme 3Minority status and language	0.148	0.471 **	0.234 *	0.469 **	0.572 **	0.166	−0.013	0.275 **	0.157	0.217 *	0.171	0.492 **	0.223 *	0.020	−0.266 **	0.051
SVI theme 4Housing type and transportation	0.172	0.330 **	0.327 *	0.100	0.212 *	0.179	−0.043	0.159	−0.129	−0.082	−0.020	0.044	0.027	−0.081	−0.223 *	−0.016

Note: yo = years old; SVI ^a^ = Social Vulnerability Index; * Correlation is significant at the 0.05 level; ** Correlation is significant at the 0.01 level.

## Data Availability

The data acquired for this study’s analyses can be found at https://covid19.ncdhhs.gov/dashboard (Accessed on 13 August 2021).

## Supplementary Materials

The following are available online at https://www.mdpi.com/article/10.3390/healthcare9121704/s1, Table S1: COVID-19 distribution for North Carolina Counties (*n* = 100).
