# Supplementary material for: Trends in COVID-19 Health Disparities in North Carolina: Preparing the Field for Long-Haul Patients
Source: Healthcare (Basel). 2021 Dec 8;9(12):1704. doi: 10.3390/healthcare9121704 (PMC8702005; doi:10.3390/healthcare9121704)
Supplement: Supplementary file 1 [file healthcare-09-01704-s001.zip › healthcare-1452031-supplementary.pdf]

Supplementary Material

Supplementary Table S1. COVID-19 distribution for North Carolina Counties (*n* = 100)

County	Cases of COVID-19 per 100,000 inhabitants	Deaths per 100,000 COVID-19 cases	Median household income (US$)	Inhabitants in poverty (%)	COVID-19 fully vaccinated per 100,000
Alamance	12185	176.9	57,963	14.6	54601.8
Alexander	12705.2	228.4	54,960	11.7	40867.3
Alleghany	10238	45.4	41,420	16.9	54377.3
Anson	11195.9	224.9	40,826	21.4	42649.1
Ashe	8889.6	167.5	41,542	14.6	50218.7
Avery	12647.1	120	45,823	16.4	50891.4
Beaufort	10549.5	218.4	49,410	17.6	51023.5
Bertie	9520.1	232.2	37,899	24.2	50129.3
Bladen	11967.6	182.6	42,260	21.2	46436.6
Brunswick	8111.4	124.4	63,712	10.2	59493.1
Buncombe	7592.5	128.1	55,448	12.2	61323.7
Burke	11983.1	182.3	47,890	18.4	43868.0
Cabarrus	11757.6	130.7	72,071	7.9	50435.9
Caldwell	12167.2	135.6	48,512	12	44250.3
Camden	6767.1	56.9	69,610	10.4	46912.7
Carteret	8099	82.5	60,058	16.2	54422.6
Caswell	10068.8	132.3	51,240	13.3	44695.6
Catawba	13294	197.3	53,688	8.7	49942.7
Chatham	7269.6	124.8	70,258	17.7	52516.4
Cherokee	10393.7	139.4	42,764	18.5	43628.5
Chowan	11257.7	262.5	47,126	14	51839.6
Clay	9117.5	164.4	47,116	19	43709.4
Cleveland	13063.1	237.5	46,012	22.3	46523.1
Columbus	13052	287.2	39,531	13.8	41325.6
Craven	9696.5	125.9	53,372	18	56300.7
Cumberland	10158.6	101.8	46,599	8.8	58214.5
Currituck	6497.7	60.7	70,699	8.9	38745.8
Dare	7034.4	27.6	63,033	15.2	68559.0
Davidson	10904.5	124.6	53,924	10.9	44467.2
Davie	10582.9	101.8	63,828	17.7	53113.5
Duplin	12027.1	245.9	44,929	14	39875.0
Durham	8681.8	77.3	65,541	21	65055.3
Edgecombe	11341.4	218.4	40,784	15.2	44365.9
Forsyth	10384.5	115.9	53,054	11.6	56685.8
Franklin	11170.5	82.9	57,371	11.6	49415.2
Gaston	13156.8	204.3	56,542	14.7	46151.3
Gates	6745.8	112.6	54,204	16.8	36914.0
Graham	8755.4	152.8	45,813	14.6	44508.9
Granville	10548.1	141.6	54,300	20.2	58744.6
Greene	12218.9	228.2	44,648	16	49152.8
Guilford	9699.2	139.8	55,820	23.8	56294.0
Halifax	11646.8	226.6	38,727	15.6	49842.0

Harnett	10055	140.6	55,619	10.6	37325.7
Haywood	8011.1	163.8	51,612	10.6	54116.9
Henderson	9619.5	143.6	61,651	23	54387.4
Hertford	8987.2	266.5	42,374	16.9	43954.0
Hoke	10058	103.8	48,420	19.2	31142.1
Hyde	13005.9	172.6	43,112	8.2	62102.5
Iredell	11746.2	135	68,308	19.3	48711.8
Jackson	9071.2	130.4	47,759	12.5	49795.2
Johnston	12269	124.4	62,835	18.8	48801.2
Jones	9881.2	302.3	46,275	14.2	47839.5
Lee	11170.5	137.2	53,114	23.1	50806.9
Lenoir	11625.2	227.3	39,947	9	50605.0
Lincoln	13322.6	103.7	70,479	14.3	46412.2
Macon	9631.5	117.8	46,279	14.6	56358.4
Madison	8019	200	50,062	20.6	53840.5
Martin	11037.7	201.3	39,413	13.6	48213.0
McDowell	12280.3	139.3	46,370	10.3	48920.4
Mecklenburg	11515.9	92.6	69,455	14.8	57884.2
Mitchell	10117.3	106.6	47,675	16.1	45068.2
Montgomery	12945.8	355.9	46,497	11.3	41173.2
Moore	10101.3	202.5	63,942	16.4	53081.9
Nash	12889.5	214.8	50,902	13	53674.5
New Hanover	9231.9	78.5	57,252	13.6	59009.4
Northampton	9781.4	305.8	57,388	21.6	43915.2
Onslow	10015.9	82.5	39,777	12.5	54333.7
Orange	6267.8	69.7	50,645	13.4	70639.0
Pamlico	9204	110.2	74,314	15.9	53819.0
Pasquotank	9027.2	220.2	52,522	14.3	47963.5
Pender	10117.7	115.9	51,245	11.5	47275.6
Perquimans	8056.6	81.9	60,405	15	43014.2
Person	9551.4	193.2	50,804	15.4	52557.6
Pitt	12366	53.2	54,553	19.2	49189.5
Polk	7758.9	155.7	52,961	12.1	42844.0
Randolph	11246	166.3	53,405	14.1	40723.3
Richmond	11927.5	237.8	50,129	25.8	44241.5
Robeson	14209.3	216.4	40,518	31.5	38418.4
Rockingham	9393.2	196.5	36,366	18.4	47447.5
Rowan	13204.9	228.8	44,686	13.9	40998.5
Rutherford	11973.2	336.3	52,051	18.5	39254.7
Sampson	13670.4	181.4	44,547	16.8	47816.0
Scotland	12139.4	259.4	45,997	28.5	45943.8
Stanly	13699.1	228.9	39,197	10.7	40878.3
Stokes	9873.1	176.9	58,303	13	43918.8
Surry	12441.1	240.4	52,356	16	48758.1
Swain	9095.4	133.2	48,637	16.1	51124.7
Transylvania	7887.5	97.7	46,075	13.1	52089.6
Tyrrell	8595.8	146.5	55,628	25.4	46240.0
Union	11579.6	99.1	37,680	12.3	50758.6
Vance	11542.5	202.3	65,712	18.5	52042.2
Wake	9047.5	70.3	40,450	8	66787.6

Warren	9337.6	100.5	37,027	21.7	51578.7
Washington	9805.4	268.4	40,157	21.3	46994.8
Watauga	9385.5	60.1	51,630	21.4	53400.9
Wayne	9866.3	199.8	45,634	18.6	47511.2
Wilkes	10936.3	170.9	45,250	15.2	44268.5
Wilson	12677.6	228.4	42,414	21.5	47942.0
Yadkin	11704.2	148.9	50,929	13.9	46255.3
Yancey	10090.1	157.7	47,664	14.2	47174.7
